# Synthesis of Atenolol-Imprinted Polymers with Methyl Methacrylate as Functional Monomer in Propanol Using Bulk and Precipitation Polymerization Method

**DOI:** 10.1155/2019/9853620

**Published:** 2019-05-19

**Authors:** Aliya Nur Hasanah, Traju Ningtias Dwi Utari, Rimadani Pratiwi

**Affiliations:** Department of Pharmaceutical Analysis and Medicinal Chemistry, Faculty of Pharmacy, Padjadjaran University, Jl. Raya Bandung Sumedang KM 21,5 Jatinangor, Sumedang, Indonesia

## Abstract

Atenolol is one of the beta-1 blocker drugs that is misused by athletes to increase their performance during competition. Therefore, it is important to analyze atenolol levels in blood selectively. The preparation method that can be used in separating atenolol in sample is molecular imprinting solid-phase extraction (MI-SPE) because it has good selectivity and sensitivity. This study aims to examine the characteristics and analytical performance of imprinted polymers synthesized from functional monomer methyl methacrylate. The stages of this study include the determination of association constants, synthesis of sorbent MI-SPE atenolol using the bulk polymerization method, and precipitation with atenolol as the template, methyl methacrylate as the functional monomer, and propanol as the porogen. The template was extracted from a polymer, and then, the adsorption ability, capacity, and selectivity of MI-SPE and finally the application of the best MI-SPE to spiked serum samples were determined. MI-SPE was also characterized by using Fourier-transform instrument infrared (FTIR) and scanning electron microscope (SEM). The result of characterization with FTIR and SEM showed that MIP made by the precipitation polymerization method was completely polymerized, more porous, and produced smaller particle size with an average value of 0.274 *μ*m. It had better analytic performances than MIP made by bulk polymerization, with affinity value 0.3607 mg/g and homogeneity value 1.3246, and good selectivity toward atenolol with imprinting factor value 22.519. Application of MI-SPE to spiked serum samples has an excellent recovery percentage of 95.46% over 0% for the nonimprinting one. Based on the result of study, MIP made by precipitation polymerization could be used to extract atenolol on serum samples toward drug analysis.

## 1. Introduction

Atenolol is an adrenoreceptor inhibitor that is selective to *β*-1, and it is used to treat hypertension, cardiac arrhythmias, angina pectoris, myocardial infarction, and other cardiovascular disorders [1]. Atenolol is often misused by athletes to improve their performance because it reduces heart rate and tremors. Thus, the use of atenolol is prohibited by the World AntiDoping Agency (WADA) and categorized as doping drugs. Therefore, monitoring levels of atenolol in blood is very important. A selective extraction method is needed because atenolol is in a complicated sample matrix [2]. The preparation method that is currently developing and has good selectivity is molecularly imprinted solid-phase extraction (MI-SPE). Molecular-imprinting is a technique for preparing polymeric materials that have prearranged structures and specific molecular recognition ability [3].

In this case, the selection of functional monomer and porogen has important role in producing molecular-specific cavities of templates. Methyl methacrylate is a functional monomer that can act as a hydrogen bond acceptor to its template [4]. Other researchers have done research on MI-SPE for atenolol [5–7], but none of them used precipitation polymerization as we used in this study, and the recoveries are below 80%. The porogen chosen in this study is propanol because it has low polarity that can reduce interference during the polymerization process and increase the number of MIP-binding sites [8].

In this study, we describe the synthesis of MIP for extraction of atenolol and for use as SPE sorbents for the selective extraction of atenolol in blood samples using two different methods. The methods are precipitation and bulk polymerization. The polymer was synthesized and characterized for the ability of the polymer to bind with atenolol selectively. The MIP was synthesized using methyl methacrylate as a functional monomer, ethylene glycol dimethacrylate as a cross linker, propanol as a porogenic solvent, and benzoyl peroxide as an initiator. The binding affinity of the binding sites in the polymer was assessed using the adsorption test. The characterization was carried out using a Fourier-transform infrared (FTIR) spectroscope and scanning electron microscope (SEM). At the end, this polymer was then used as an extraction material of atenolol from the spiked blood serum.

## 2. Materials and Methods

### 2.1. Materials

Acetonitrile was purchased from Fisher Chemical; atenolol, metoprolol, and propranolol were purchased from TCI. Acetic acid 96%, benzoyl peroxide (BPO), methanol, potassium bromide, and propanol were purchased from Merck. Ethylene glycol dimethacrylate (EGDMA) and methyl methacrylate (MMA) were purchased from Sigma-Aldrich. All chemicals used were commercially available and were of analytical grade. Methanol used was of HPLC gradient grade. Methyl methacrylate was purified before being used with aluminium chloride.

### 2.2. Determination of Association Constant of Monomer Template

Atenolol 2 × 10^−5^ M was diluted in propanol. 2.5 ml of the solution was measured using a UV-Vis spectrophotometer. Methyl methacrylate solution of 0.01 M was added gradually from 10 *μ*l, 20 *μ*l, 30 *μ*l, 40 *μ*l, 50 *μ*l, 100 *μ*l, and so on, until there was no significant increase in the absorbance value. The results are plotted on a graph between 1/[*G*] and 1/Δ*Y*. The constant association was calculated using Benesi–Hildebrand equation [9].

(1) $$\begin{array}{c} \frac{1}{\Delta Y}=\frac{1}{Y\Delta \text{HGKa}\left[G\right]}+\;\frac{1}{Y\Delta \text{HG}}, \end{array}$$

where Δ*Y* is the change in absorbance, *Y*ΔHG is the change in absorbance at the end of titration, and [*G*] is the concentration added [10].

### 2.3. Synthesis of Atenolol-Imprinted Polymer Using Bulk Polymerization

A prepolymerization solution consisting of 0.263 g atenolol, 400 *μ*l MMA, 3.77 ml EGDMA, 250 mg BPO, and 5 ml propanol was prepared in a vial. The reaction molar ratio of the template, functional monomer, and cross linker for the preparation of MIP was 1 : 4 : 20. The solution was sonicated for 20 minutes to remove oxygen. The atenolol-MIP was placed in an oven at 70°C for 1 hour and moved to water bath and kept at 70°C for 18 hours for polymerization. The resulting bulk polymers were ground and sieved using mesh 60, washed using 20 ml methanol, and dried at 50°C. The nonimprinted polymer (NIP) was prepared simultaneously under the same condition without the addition of the template [8–11].

### 2.4. Synthesis of Atenolol-Imprinted Polymer Using Precipitation Polymerization

A prepolymerization solution consisting of 0.133 g atenolol, 200 *μ*l MMA, 1.885 ml EGDMA, 750 mg BPO, and 175 ml propanol was prepared in a glass vial. The reaction molar ratio of the template, functional monomer, and cross linker for the preparation of MIP was 1 : 4 : 20. The solution was sonicated for 20 minutes to remove oxygen. The atenolol-MIP was placed in an oven at 70°C for 1 hour and moved to water bath shaker and kept at 70°C for 18 hours for polymerization. The resulting bulk polymers were washed using 40 ml methanol and dried at 50°C. The nonimprinted polymer (NIP) was prepared simultaneously under the same condition without the addition of template [9].

### 2.5. Extraction of Template

The Soxhlet apparatus was used for template removal from the synthesized MIP using methanol and acetic acid (9 : 1) for 24 hours. Then, the polymers were washed using 20 ml methanol and water and dried at 50°C for 18 hours.

MIP was monitored using 20 mg MIP diluted in 5 ml methanol, triplicate. Then, MIP was sonicated for 5 minutes and set aside for 24 hours. The extraction process is complete when the liquid leaching results in MIP no longer contain the template when monitored using a UV-Vis spectrophotometer [11].

### 2.6. Evaluation of Binding Ability

Twenty milligrams of MIP in vial was incorporated into 5 ml atenolol at a concentration of 5 ppm in various solutions such as methanol, acetonitrile, and methanol : acetonitrile (1 : 1), triplicate. The vials were shaken using a shaker at 120 rpm for 3 hours at room temperature to reach equilibrium and then filtered. The filtrate was measured by using a UV-Vis spectrophotometer. The evaluation of NIP was carried out by the same procedure as that for MIP [11].

### 2.7. Evaluation of Binding Capacity

Five milliliters of selected solvent from evaluation 2.6 containing 2.5; 5; 7.5; 10 ppm of atenolol was added to 20 mg of MIP in vials, triplicate. The vials were shaken using a shaker at 120 rpm for 3 hours at room temperature and then filtered. The filtrate was measured by using a UV-Vis spectrophotometer. The results are plotted for isotherm Freundlich adsorption curve. The adsorption curve of NIP was plotted by the same procedure as that for MIP [11, 12].

### 2.8. Determination of Selectivity

Atenolol, metoprolol, and propranolol were used to determine the relative selectivity of MIP. 5 ml of selected solvent from evaluation 2.6 containing 5 ppm of atenolol; metoprolol; propranolol was added to 20 mg of MIP in vials, triplicate. The vials were shaken using a shaker at 120 rpm for 3 hours at room temperature and then filtered. The filtrate was measured by using a UV-Vis spectrophotometer. The evaluation of NIP was carried out by the same procedure as that for MIP. The imprinting factor was calculated using the following equation:

(2) $$\begin{array}{c} K_{\text{D}}=\frac{\left(C_{\text{i}}-C_{\text{f}}\right)V}{C_{\text{f}}m},\\ \text{IF}=\frac{K_{\text{D}}\text{ MIP}}{K_{\text{D}}\text{ NIP}}, \end{array}$$

where *K*_D_ is the distribution coefficient; *C*_i_ and *C*_f_ are the concentration of atenolol before and after the adsorption experiments, respectively; *V* is the volume of solution containing atenolol; and *W* is the weight of the polymer [13, 14].

### 2.9. Application of the Polymer in Serum Samples

The blood serum is obtained by centrifugation of blood at a speed of 5000 rpm for 5 minutes; then the supernatant is collected. The blood serum is spiked with 2 ppm atenolol in water. The spiked serum is passed into MIP-SPE and NIP-SPE. The SPE system is conditioned with methanol : acetonitrile (1 : 1) 3 × 1 mL, washing solvents using acetonitrile, and elution using methanol : trifluoroacetic acid 0.05% (99 : 1) 3 × 1 mL. The elution results were then analyzed by HPLC using the mobile phase of methanol : water + triethylamine 0.05% which was adjusted to pH 3 with phosphoric acid (15 : 85).

### 2.10. Characterization of Atenolol-Imprinted Polymer

The chemical structure of MIP and NIP samples was characterized by FTIR spectroscopy (IRPrestige-21, Shimadzu). Samples were ground and pressed into KBr plates. The analysis was performed between 400 and 4000 cm^−1^. The surface morphology was analyzed by SEM [11, 15, 16].

## 3. Results and Discussion

### 3.1. Determination of Association Constant of Monomer Template

Before the polymerization step, the association constant was determined to know the ability of MMA functional monomer to bind with atenolol to form a stable complex in prepolymerization solution with the titration method using a UV-Vis spectrophotometer [17].

**Figure 1:**
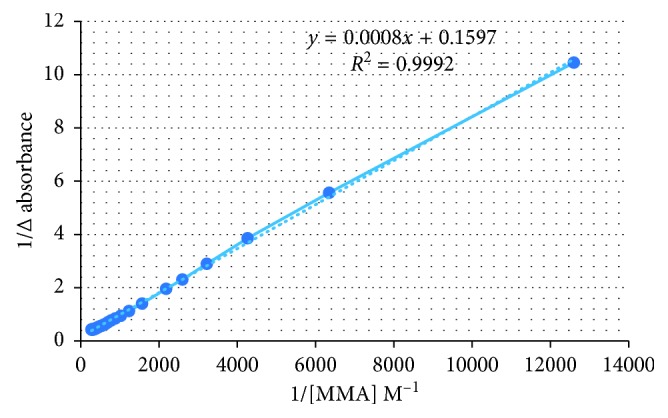
Relationship between 1/(methyl methacrylate) to 1/Δabsorbance.

The association constant was 199.625 M^−1^, calculated by Benesi–Hildebrand equation (Figure 1). The higher the value of the association constant, the more stable the complex that occurs during polymerization and the better the imprinting effect [18, 19].

### 3.2. Synthesis of Atenolol-Imprinted Polymer Using Bulk and Precipitation Polymerization

The purpose of the synthesis by two methods is to see the effectiveness of each polymer produced. In molecular-imprinting processes, the selection of the functional monomer is an important factor that affects the binding affinity and specificity of the imprinted polymer. The formulations were prepared by the bulk and precipitation polymerization method using MMA as the monomer, BPO as the initiator, and EGDMA as the cross linker. The ratio of the monomer affected the particle sizes and % yields of the obtained MIP and NIP [20].

### 3.3. Extraction of Template

The purpose of extraction was to remove atenolol groups that bind to polymers and to form cavities that were complementary to atenolol [18]. Atenolol is soluble in methanol, so it was used to extract the template. Acetic acid was added to disrupt the hydrogen bond between atenolol and the functional monomer MMA to facilitate the removal of atenolol [12, 21].

### 3.4. Evaluation of Binding Ability

In order to know the binding ability and to find out the optimum conditions for the template to be recognized by the MIP that is being prepared, a standard solution of atenolol of 5 ppm was initially prepared in various solvents such as methanol, acetonitrile, and methanol : acetonitrile (1 : 1). The filtrate that indicated the amount of unbound analyte was measured. The atenolol-binding ability of MIPs was investigated and compared with that of NIPs [15].

**Figure 2:**
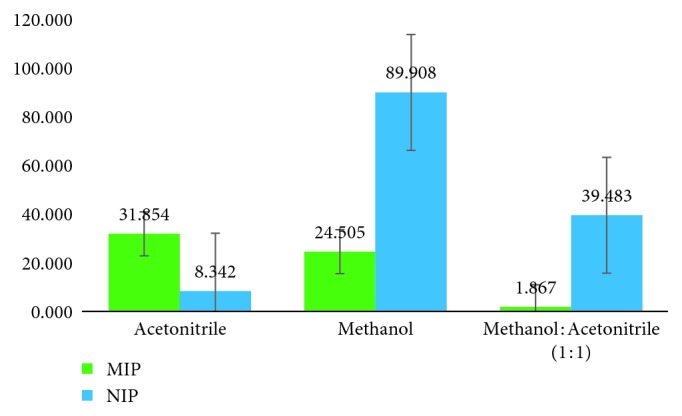
Binding ability MMA propanol polymer bulk method (*n* = 3).

**Figure 3:**
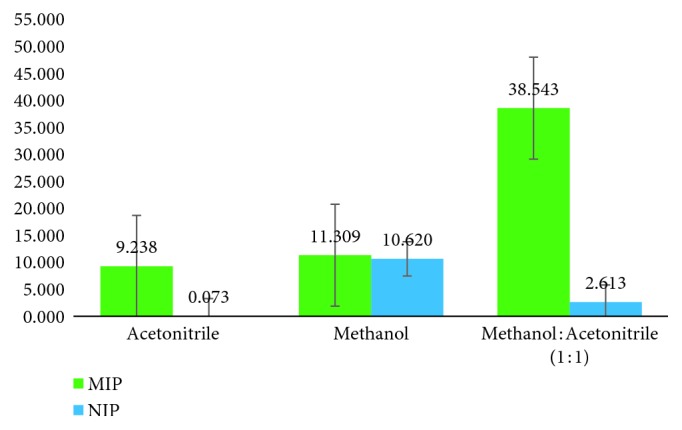
Binding ability MMA propanol polymer precipitation method (*n* = 3).

From Figure 2, it is known that the MIP synthesized using the bulk polymerization method can bind with atenolol in acetonitrile, with 31.854% of binding. However, NIPs in other solvents such as methanol and methanol : acetonitrile (1 : 1) showed a higher percent of binding, 89.908% and 39.483%, respectively. This suggests that NIPs swelled better in these solvents. From Figure 3, the MIP synthesized using the precipitation polymerization method can bind atenolol in methanol : acetonitrile (1 : 1), with 38.543% of binding. This showed that acetonitrile has the ability to lead atenolol properly to the binding site in MIP [13, 22, 23].

### 3.5. Evaluation of Binding Capacity

This evaluation is used to study the affinity between MIP and analyte target [23]. The relationship between the amount of analyte bound to the adsorbent (*B*) and the amount of free analyte (*F*) is illustrated in the following Freundlich isotherm equation:

(3) $$\begin{array}{c} \log\;B=m\;\log\;F+\log\;a\text{.} \end{array}$$

The value of *m* indicates the homogeneity index; when it approaches 1, the sorbent is more homogeneous, and when it approaches 0, the sorbent is more heterogeneous [13]. The value of *a* indicates sorbent affinity; the higher the affinity obtained, the more the capacity of the sorbent to bind to the analyte target [24, 25].  Table 1 shows the binding capacity value of each MIP and NIP. The MIP that was synthesized using the precipitation method has a homogeneity value close to 1, which is 1.3426, and an affinity value of 0.3607. It has better *a* and *m* values than the MIP that was synthesized by bulk polymerization, which indicates better imprinting effect.

### 3.6. Determination of Selectivity

The selectivity for atenolol of MIP was investigated by determining its binding ability compared with other *β*-blocker drugs such as metoprolol and propranolol. A testing solution containing 5 ppm of each drug (atenolol, metoprolol, and propranolol) was prepared [17]. MIP and NIP were prepared 20 mg in each vial. Solution and MIP were combined in a vial then shaken using a shaker to reach equilibrium. The filtrate was measured using a UV-Vis spectrophotometer. The distribution coefficient (*C*_D_) and imprinting factor (IF) were calculated. The results (Tables 2 and 3) showed that atenolol can bind with MIP synthesized by the precipitation method with a distribution coefficient of 156.864 ml/g, which is higher than that of NIP which was 6.966 ml/g. The imprinting factor of MIP was 22.519. This indicates that the MIP that was synthesized by the precipitation method had a higher selectivity for atenolol than the other *β*-blocker drugs. Thus, it was selective for atenolol.

### 3.7. Application of the Polymer in Serum Samples

The polymer made by precipitation polymerization has better analytical performances compared to bulk. Based on this result, a 100 mg polymer made by precipitation polymerization then was packed into cartridges and used as an SPE material over the spiked blood serum. The imprinting polymer gain recovery percentage was 95.46% ± 3.44% compared to nonimprinted 0.00% ± 0.00%. This result showed an excellent imprinting effect and was linear to the selectivity result which had an imprinting factor value of 22.519 compared with NIP. All experiments were done using the same cartridges (over 20 cycles), and the results are still in the acceptance range (SD below 5%). The result of this polymer is better than that of the one in the previous study [7] and fits with criteria recoveries of analyte from biological fluid Table 4.

### 3.8. Characterization of Atenolol-Imprinted Polymer

**Figure 4:**
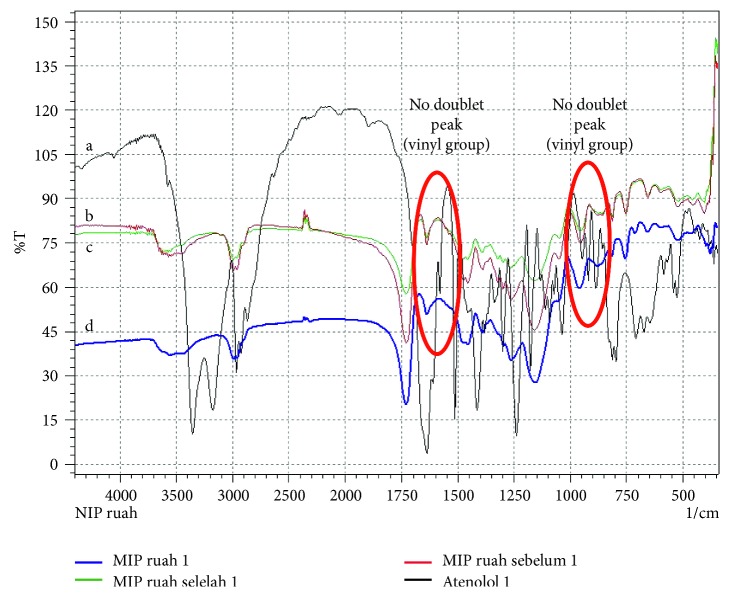
FTIR spectrum: (a) atenolol; (b) MIP by bulk before extraction; (c) MIP by bulk after extraction; (d) NIP by bulk.

**Figure 5:**
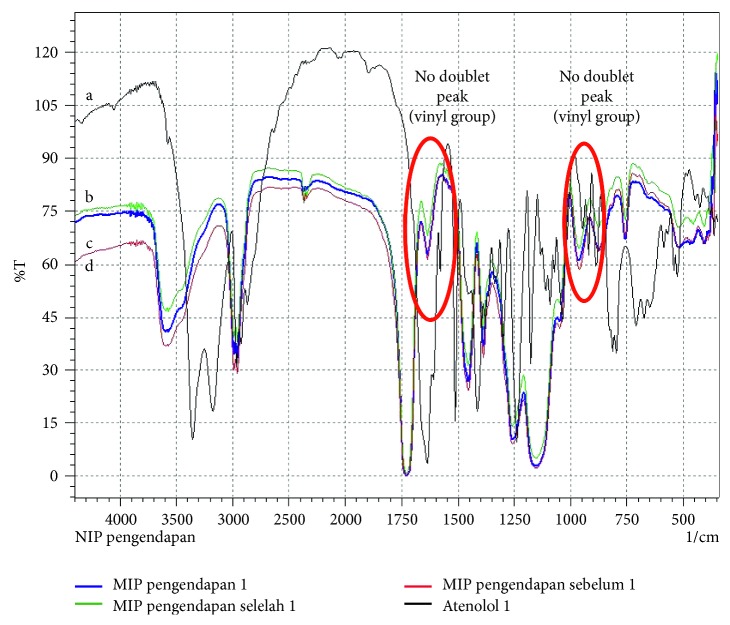
FTIR spectrum: (a) atenolol; (b) MIP by precipitation after extraction; (c) NIP by precipitation; (d) MIP by precipitation before extraction.

The FTIR spectrums of the MIP by bulk and precipitation polymerization are presented in Figures 4 and 5. The complete polymerization process was characterized when the doublet peak (vinyl group, H_2_C = CH-) was absent at the wavenumber 1600 cm^−1^, 1660 – 1635 cm^−1^, 990 ± 5 cm^−1^, and 910 ± 5 cm^−1^ [26].

**Figure 6:**
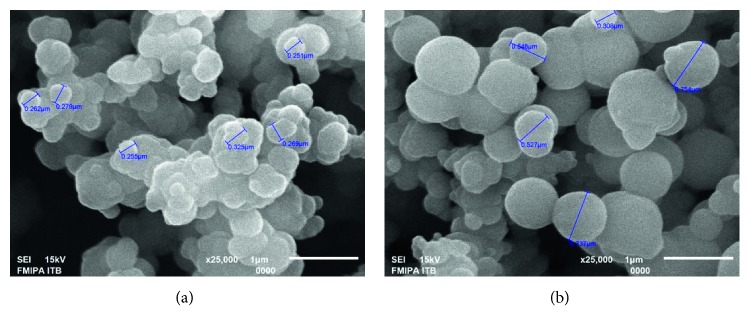
SEM images of (a) MIP by precipitation polymerization and (b) NIP by precipitation polymerization.

The morphologies and particle size of MIP and NIP that were synthesized by precipitation polymerization were determined using SEM and the results are shown in Figure 6. The SEM images of the NIP revealed a spherical and smooth surface without evidence of collapsed particles, whereas those of the MIP revealed spheres with small pores on the surface [17]. MIP had smaller particles; it suggested that the template compound has an important influence on the particle growth during the precipitation polymerization [20].

## 4. Conclusions

The molecularly imprinted polymer of atenolol with MMA as the functional monomer in propanol using the precipitation polymerization synthesis method had better analytic performances than MIP that was synthesized using the bulk polymerization method. The value of affinity was 0.3607 mg/g, and the value of homogeneity was 1.3246, selective toward atenolol with an imprinting factor of 22.519. Application of MI-SPE to spiked serum samples has an excellent recovery percentage of 95.46% over 0% for the nonimprinting one. The results of characterization also showed that the MIP that was synthesized using the precipitation method had small-sized homogenic particles.

## Figures and Tables

**Table 1:** Binding capacity MMA propanol bulk and precipitation method (*n* = 3).

Value	MMA propanol bulk		MMA propanol precipitation	
	MIP	NIP	MIP	NIP
*M*	−0.1909	0.102	1.3246	2.3672
*a* (mg/g)	0.07	0.0872	0.3607	0.0028
*R* ^2^	0.0198	0.0182	0.9547	0.8061

**Table 2:** Selectivity of MMA propanol polymer by bulk polymerization (*n* = 3).

Analyte		Atenolol	Propranolol	Metoprolol
*K*_D_ (ml/g)	MIP	115.385	218.624	0.170
	NIP	22.135	332.559	0.022
Imprinting factor		5.213	0.657	7.869

**Table 3:** Selectivity of MMA propanol polymer by precipitation polymerization (*n* = 3).

Analyte		Atenolol	Propranolol	Metoprolol
*K*_D_ (ml/g)	MIP	156.863	97.32	334.172
	NIP	6.966	176.443	144.475
Imprinting factor		22.519	0.553	2.313

**Table 4:** Result of application of the polymer in serum samples compared to previous study [7].

	Polymer MMA made by precipitation polymerization	Polymer from previous study [7]
Imprinting factor (IF)	22.519	4.18
Recoveries	95.46%	74.5–75.3%

## Data Availability

The data used to support the findings of this study are included within the article.
